# Impact of obesity in colorectal endoscopic submucosal dissection: single-center retrospective cohort study

**DOI:** 10.1186/s12876-021-01652-5

**Published:** 2021-02-16

**Authors:** Jun Tachikawa, Hideyuki Chiba, Naoya Okada, Jun Arimoto, Keiichi Ashikari, Hiroki Kuwabara, Michiko Nakaoka, Takuma Higurashi, Toru Goto, Atsushi Nakajima

**Affiliations:** 1 Department of Gastroenterology, Omori Red Cross Hospital, 4-30-1, Chuo, Ota-Ku, Tokyo, 143-0024 Japan; 2 Department of Gastroenterology and Hepatology, Yokohama City University Graduate School of Medicine, 3-9, Fukuura, Kanazawa-Ku, Yokohama, 236-0004 Japan

**Keywords:** Colorectal cancer, Endoscopic submucosal dissection, Obesity, Body mass index

## Abstract

**Background:**

When performing colorectal endoscopic submucosal dissection (ESD) in obese patients, technically difficult cases are sometimes experienced because of difficulty with the insertion of the colonoscope, poor scope maneuverability, or an abundance of fat tissue in the submucosal layer. Since the association between obesity and colorectal ESD has not been investigated, we evaluated the clinical impact of obesity in patients who underwent colorectal ESD.

**Methods:**

We retrospectively reviewed 535 patients who underwent colorectal ESD between April 2012 and February 2019. Patients were divided into three groups based on their body mass index (BMI): a control group (BMI < 25 kg/m^2^), an overweight group (25 kg/m^2^ ≤ BMI < 30 kg/m^2^), and an obese group (BMI ≥ 30 kg/m^2^), and the short-term clinical outcomes were analyzed to assess the safety and difficulty of colorectal ESD.

**Results:**

No significant difference in the procedure times, *en bloc* resection rates, pathological diagnoses, or complications were seen among the groups. While the amount of sedative per body weight was significantly lower in the group with a higher BMI (flunitrazepam: 1.75 × 10^−2^ [1.27 × 10^−2^–2.34 × 10^−2^] mg/kg vs. 1.48 × 10^−2^ [1.08 × 10^−2^–2.03 × 10^−2^] mg/kg vs. 1.16 × 10^−2^ [0.98 × 10^−2^–1.54 × 10^−2^] mg/kg, *P* < 0.001; pethidine: 0.63 [0.55–0.72] mg/kg vs. 0.50 [0.46–0.56] mg/kg vs. 0.39 [0.32–0.45] mg/kg, *P* < 0.001), a reduction in percutaneous arterial oxygen saturation occurred significantly more frequently in the group with a higher BMI (123 [30.2%] vs. 43 [43.9%] vs. 10 [55.6%], *P* = 0.005). When the procedures were performed by trainees, the number of cases that required a procedure time of longer than 90 min was significantly larger in the group with a higher BMI (27 [10.8%] vs. 14 [21.9%] vs. 3 [25.0%], *P* = 0.033).

**Conclusions:**

This study showed that colorectal ESD could be performed safely and effectively in obese patients. However, ESD in obese patients requires attention, particularly to changes in respiratory conditions.

## Background

The number of obese people is increasing worldwide and obesity has been shown to have various effects on the clinical outcomes of a variety of diseases. Several studies have reported an association between obesity and surgery, suggesting that the risks of peri- and post-operative complications associated with surgery for colorectal cancer are higher for obese patients [1–6]. In obese patients, securing an operative field during surgery can be difficult because of a thick abdominal wall and abundance of visceral fat. In addition, since fat is a very poor conductor of electricity, unnecessary invasion to other organs is likely to occur. Therefore, obesity has been associated with longer operation times, longer hospital stays, and postoperative complications such as cardiovascular disease, pulmonary embolism, and wound infections. However, a significant difference in mortality has not been observed.

**Fig. 1:**
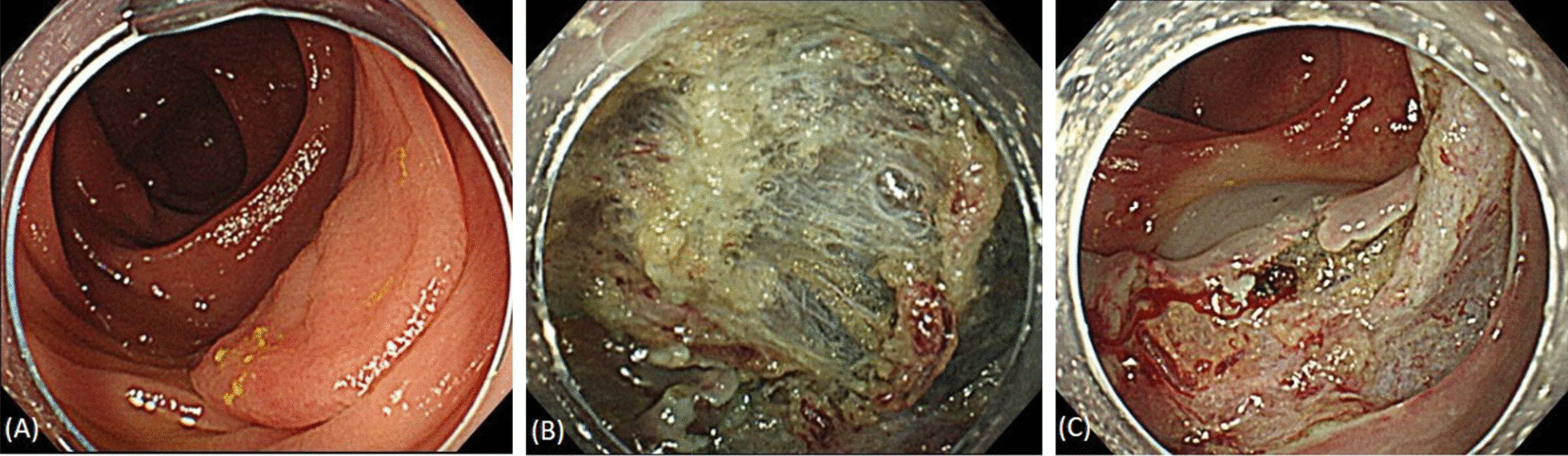
Lesion with abundant fat tissue in the submucosal layer. **A** Non-granular laterally spreading tumor that was 34 mm in size and located in the ascending colon of an obese patient whose body mass index was 26.7 kg/m^2^. **B** Abundant fat tissue in the submucosa. **C** Fat tissue which was also found on the ulcer after resection

On the other hand, colorectal endoscopic submucosal dissection (ESD) has become widespread as a treatment for early colorectal cancer. Although ESD for large lesions has become possible, mainly in Japan and Asia [7], such procedures remain challenging in Western countries. Several reports have described the difficulties of treating such lesions, including fibrosis and poor scope manipulation [8, 9], but no reports have examined the relationship between ESD and obesity. According to a Korean study, gastric ESD in obese patients was associated with a longer procedure time; however, no differences in the complication rates and *en bloc* resection rates were seen [10]. In that study, fatty tissue in the submucosal layer was suggested to have complicated the ESD procedures. When abundant fat tissue is present in the submucosal layer, securing a field of view can be difficult, and the submucosal tissue is more likely to be burnt, potentially resulting in poor cauterization against bleeding (Fig. 1) [11, 12]. In addition, when performing colorectal ESD in obese patients, technically difficult cases are sometimes experienced because of difficulty with the insertion of the colonoscope or poor scope maneuverability. However, the impact of obesity on the outcomes of patients undergoing colorectal ESD has not yet been directly assessed. In this study, we clarified the clinical impact of obesity on the short-term outcomes of ESD for colorectal neoplastic lesions not only during hospitalization, but also with respect to delayed events after ESD.

## Methods

### Patients

We retrospectively reviewed 535 patients who underwent colorectal ESD between April 2012 and February 2019 at our hospital. Patients whose body mass index (BMI) was unknown, in whom ESD was discontinued, and who presented with neuroendocrine tumors were excluded. The remaining 523 patients were divided into three groups based upon their BMI according to the World Health Organization’s criteria for obesity: control group (BMI < 25 kg/m^2^), overweight group (25 kg/m^2^ ≤ BMI < 30 kg/m^2^), and obese group (BMI ≥ 30 kg/m^2^) [13]. Informed consent was obtained from each patient included in the study.

### ESD Procedures

The ESD procedure were performed using a single-channel endoscope (PCF-Q260 JI, GIF-Q260J; Olympus Co., Tokyo, Japan) with a transparent attachment (D-201-11802; Olympus Co.), and carbon dioxide was used for insufflation. The tumor resection devices were the FlexKnife® (KD-630L; Olympus Co.) or the DualKnife® (KD-650L; Olympus Co.) with a VIO 300D high-frequency generator (ERBE, Tübingen, Germany), and the solution for local injection was a mixture of 0.4% sodium hyaluronate and indigo carmine with diluted epinephrine. In all the cases, patients were dosed with 2 L of polyethylene glycol electrolyte solution to prepare the bowel prior to ESD. For conscious sedation, flunitrazepam (0.2–0.5 mg) and pethidine (17.5–35 mg) were administered for conscious sedation at the beginning of the procedure and added as appropriate. Glucagon or scopolamine was injected intravenously to inhibit peristalsis of the colon during the procedure. Blood pressure, heart rate, electrocardiography, and oxygen saturation were monitored during the procedure. The procedures were performed by one expert who had performed > 200 colorectal ESD procedures (as of 2012) and 6 trainees, each of who had performed > 30 gastric ESD procedures and < 100 colorectal ESD procedures. The expert was always present to supervise and guide the ESD procedures. The expert took over the operation from the trainee when any of the following conditions were met: (1) the total procedure time exceeded about 2 h; (2) perforation occurred or was likely to have occurred; (3) continuation of the procedure seemed difficult; or (4) a better resection method needed to be taught. The mucosal incision was made using the End-cut I mode (effect 2, duration 2, interval 2), and the submucosal dissection was performed primarily in the End-cut I mode and sometimes in swift coagulation mode (effect 2, 45 W) for submucosal areas rich in vessels or fat tissue [9].

### Definitions and outcome measurements

The ESD procedure time was defined as the elapsed time from the start of the local injection until the complete removal of the lesion. Based on previous reports, we defined a “prolonged procedure” as one that required more than 90 min [14–16]. The degree of submucosal fibrosis was defined according to previous reports as follows: F0, no fibrosis, manifesting as a blue transparent layer; F1, mild fibrosis, appearing as a white web-like structure in the blue submucosal layer; and F2, severe fibrosis, appearing as a white muscular structure without a blue transparent layer in the submucosal layer [17]. The specimen size was defined as the major diameter of the resected sample. Systolic blood pressure fluctuations, bradycardia, and hypoxemia were defined as occurring if there was a change in blood pressure, heart rate, or percutaneous arterial oxygen saturation (SpO_2_) at any point during treatment. We defined delayed bleeding after ESD as a decrease in the hemoglobin level by at least 2 g/dL or more below the most recent preoperative level, and/or the necessitation of blood transfusion and/or marked hematochezia [18, 19]. We defined a delayed event after colorectal ESD as an adverse event other than bleeding and perforation if it occurred within 30 days after ESD was performed.

### Histopathological assessment

All specimens were cut into 2 mm slices and stained with hematoxylin and eosin. The specimens were examined by experienced pathologists to determine the histological type, depth of invasion, the presence/absence of lymphatic invasion and vascular involvement, and the lateral and vertical resection margins. *En bloc* resection was defined as the removal of the tumor in a single piece, and complete resection was defined as en bloc resection with vertical and horizontal margin negative. Patients were defined as having undergone curative resection when the following criteria based on the Japanese Classification for Cancer of the Colon and Rectum were met: lateral and vertical margins were free of tumor cells, the tumor was an intramucosal carcinoma or a carcinoma with mild submucosal invasion (invasion depth of less than 1000 μm), and lymphatic invasion, vascular involvement, grade 2 or 3 tumor budding, or a poorly differentiated tumor component were absent [20].

### Statistical analyses

All the variables were tested for normality using the Shapiro–Wilk test; nonparametric tests were used for data that were not normally distributed. Continuous data were presented as the median and interquartile ranges, and categorical data were presented as quantities and proportions. For the statistical analyses, we used a chi-squared test and a Kruskal–Wallis test. All the analyses were performed with SPSS 23 for Windows. *P* values ≤ 0.05 were considered to denote statistical significance.

### Ethics

The study was conducted in accordance with the principles laid down in the Declaration of Helsinki, and with the approval of the Institutional Review Board of Omori Red Cross Hospital (No. 19-31).

## Results

### Baseline characteristics

**Fig. 2:**
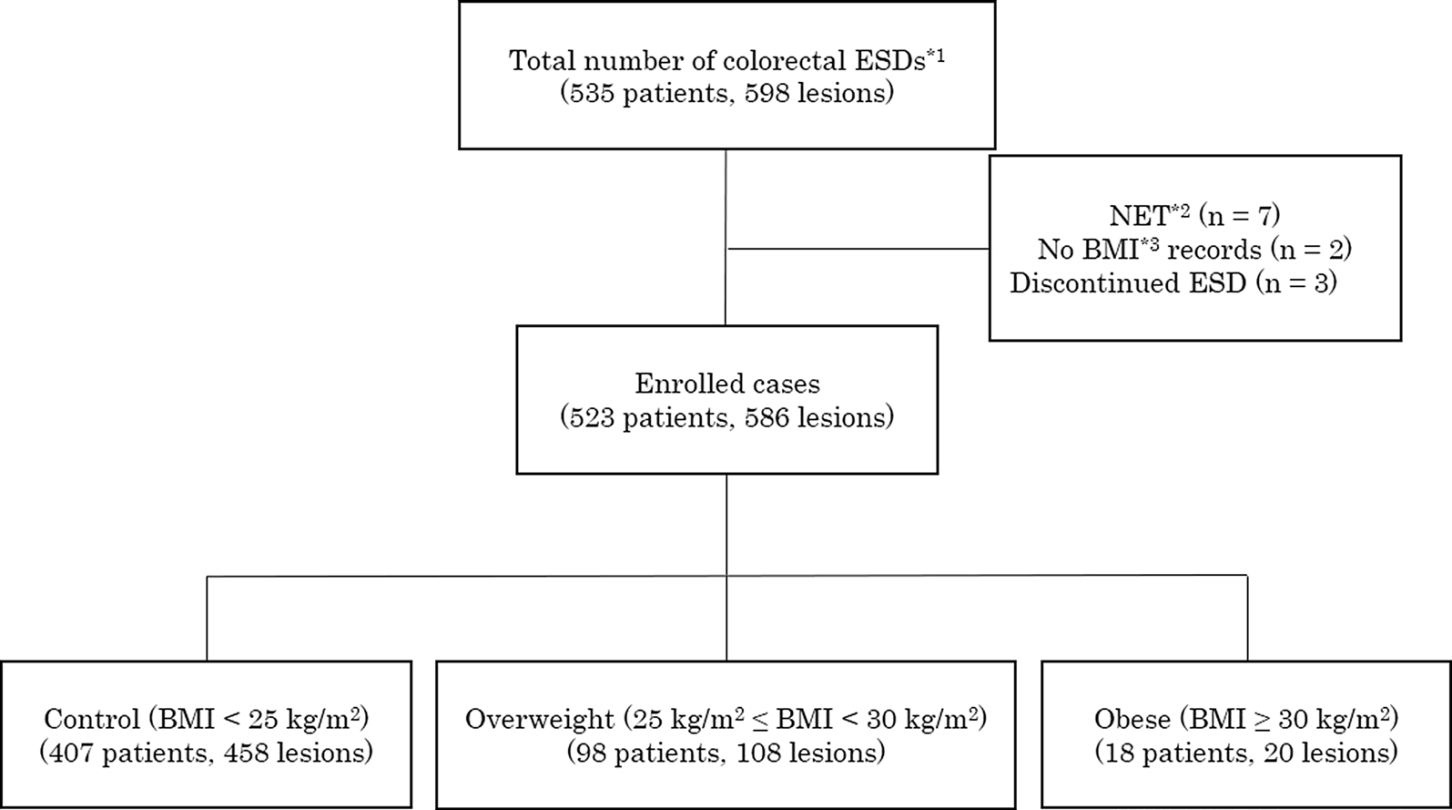
Patients who had underwent colorectal ESD and did not meet the exclusion criteria were divided into three groups according to their BMI. *1 Endoscopic submucosal dissection. *2 Neuroendocrine tumor. *3 Body mass index

A total of 535 patients who received ESD for a colon tumor during the study period were evaluated. The total number of lesions was 598. Patients who were treated for a neuroendocrine tumor (n = 7), did not have BMI records (n = 2) or whose ESD was discontinued (n = 3) were excluded. The remaining 523 patients (586 lesions) were analyzed in our study (Fig. 2). The baseline characteristics of the patients and their lesions are listed in Table 1. The median age (interquartile range) was 70.0 (62.5–77.0) years, males comprised 57.4% (n = 300), and the median BMI was 22.8 (20.3–24.8) kg/m^2^. Patients whose BMI was less than 25 kg/m^2^ were classified as belonging to the control group, those with a BMI of 25 kg/m^2^ or more and less than 30 kg/m^2^ were classified as belonging to the overweight groups, and those with a BMI of 30 kg/m^2^ or more were classified as belonging to the obese group [13]. No significant difference in median age or the proportion of males: females was seen among the groups. Furthermore, no significant differences in the number of patients with comorbidities and taking antithrombotic medication were seen. No significant difference in the cecal insertion time during the preoperative colonoscopy was seen. Other patient characteristics did not differ among the groups. The lesion characteristics, locations, macroscopic types, and pathologies were similar among the groups.

**Table 1:** Baseline characteristics of the patients and lesions

	Total	Control	Overweight	Obese	P value
		(BMI < 25 kg/m^2^)	(25 kg/m^2^ ≤ BMI < 30 kg/m^2^)	(BMI ≥ 30 kg/m^2^)	
Patients (*n*)	523	407	98	18	
Age (median [IQR^*1^])	70.0 (62.5–77.0)	71.0 (63.0–77.0)	69.5 (61.5–75.0)	67.5 (59.3–80.0)	0.354
Sex, male (*n*)	300 (57.4%)	227 (55.8%)	64 (65.3%)	9 (50.0%)	0.188
BMI^*2^ (kg/m^2^, median [IQR])	22.8 (20.3–24.8)	21.7 (19.8–23.4)	26.3 (25.6–27.3)	32.4 (31.2–34.0)	< 0.001
Cecal insertion time^*3^ (min, median [IQR])	5.5 (4.0–7.0)	6.0 (4.0–7.5)	5.0 (3.0–6.0)	5.0 (4.0–5.25)	0.086
Comorbidities (*n*)					
Hypertension	236 (45.1%)	174 (42.8%)	53 (54.1%)	9 (50.0%)	0.118
Diabetes	66 (12.6%)	46 (11.3%)	18 (18.4%)	2 (11.1%)	0.164
Cardiovascular disease	17 (3.3%)	14 (3.4%)	2 (2.0%)	1 (5.6%)	0.668
Cerebrovascular disease	53 (10.1%)	42 (11.5%)	7 (7.1%)	4 (22.2%)	0.145
Liver cirrhosis	9 (1.7%)	7 (1.7%)	2 (2.0%)	0 (0.0%)	0.829
Chronic renal failure	15 (2.9%)	14 (3.4%)	1 (1.0%)	0 (0.0%)	0.331
Respiratory disease^*4^	21 (4.0%)	19 (4.7%)	2 (2.0%)	0 (0.0%)	0.334
Antithrombotic medication (*n*)	90 (17.2%)	71 (17.4%)	15 (15.3%)	4 (22.2%)	0.747
Lesion (*n*)	586	458	108	20	
Location (*n*)					0.809
Cecum	84 (14.3%)	66 (14.4%)	13 (12.0%)	5 (25.0%)	
Ascending	141 (24.1%)	108 (23.6%)	28 (25.9%)	5 (25.0%)	
Transverse	131 (22.4%)	100 (21.8%)	26 (24.1%)	5 (25.0%)	
Descending	47 (8.0%)	35 (7.6%)	11 (10.2%)	1 (5.0%)	
Sigmoid	84 (14.3%)	66 (14.4%)	15 (13.9%)	3 (15.0%)	
Rectum	99 (16.9%)	83 (18.1%)	15 (13.9%)	1 (5.0%)	
Macroscopic type (*n*)					0.795
Protruded	99 (16.9%)	78 (17.0%)	17 (15.7%)	4 (20.0%)	
Elevated	482 (82.2%)	375 (81.9%)	91 (84.3%)	16 (80.0%)	
Depressed	5 (0.9%)	5 (1.1%)	0 (0.0%)	0 (0.0%)	
Pathologycal diagnosis (*n*)					0.636
Adenoma	367 (62.6%)	293 (64.0%)	64 (59.3%)	10 (50.0%)	
Tis	165 (28.2%)	123 (26.9%)	34 (31.5%)	8 (40.0%)	
T1	54 (9.2%)	42 (9.2%)	10 (9.3%)	2 (10.0%)	

^*^1 Interquartile range

^*^2 Body mass index

^*^3 Cecal insertion time of the colonoscopy for pre-ESD observation

^*^4 interstitial lung disease or chronic obstructive pulmonary disease

### Clinical outcomes

Table 2 presents the clinical outcomes of colorectal ESD according to the body mass index groups. *En bloc* resection was achieved in 585 (99.8%) patients, complete resection was achieved in 579 (98.8%) patients, and curable resection was achieved in 573 (97.8%) patients. No significant differences in the *en bloc* and curable resection rates were seen among the groups. The median specimen size was 32.0 (24.0–40.0) mm, and the median lesion size was 24.0 (20.0–30.0) mm. No significant differences in these parameters were seen among the groups. In the pathological results for the resected specimens, the frequencies of vertical margin positivity, horizontal margin positivity, lymphovascular invasion, tumor budding, and the submucosal invasion of tumor cells did not differ significantly among the groups. The median procedure time was 31.0 (20.0–53.0) min, and 62 (10.4%) cases required a procedure time of longer than 90 min. No significant difference in procedure time was seen among the groups. Regarding monitoring during the procedure, no significant difference in a systolic blood pressure variability of more than 50 mmHg or the incidence of bradycardia was seen. The number of cases with hypoxemia differed significantly among the groups, with a higher BMI associated with a greater likelihood of hypoxemia (123 [30.2%] vs. 43 [43.9%] vs. 10 [55.6%], *P* = 0.005). Nevertheless, the amount of sedative per body weight was significantly higher in the group with a lower BMI (flunitrazepam: 1.75 × 10^−2^ [1.27 × 10^−2^–2.34 × 10^−2^] mg/kg vs. 1.48 × 10^−2^ [1.08 × 10^−2^–2.03 × 10^−2^] mg/kg vs. 1.16 × 10^−2^ [0.98 × 10^−2^–1.54 × 10^−2^] mg/kg, *P* < 0.001; pethidine: 0.63 [0.55–0.72] mg/kg vs. 0.50 [0.46–0.56] mg/kg vs. 0.39 [0.32–0.45] mg/kg, *P* < 0.001). Perforation was observed in four cases and bleeding in seven cases, but no significant difference was seen among the groups. All the patients with delayed bleeding were treated with endoscopic hemostasis. Three of the patients with perforation were treated with endoscopic closure, and one underwent emergency surgery for delayed perforation. After ESD, a few delayed events occurred. One patient developed a pulmonary embolism and one developed sick sinus syndrome in the overweight group, while one patient developed a pulmonary embolism and one developed subarachnoid hemorrhage in the control group. During the follow-up period (range, 2–87 months), none of the patients died as a result of colorectal cancer, although 10 patients died as a result of other diseases (5 from lung cancer, 1 from malignant lymphoma, and 4 from unknown causes).

**Table 2:** Clinical outcomes of colorectal ESD according to body mass index groups

	Total	Control (BMI^*8^ < 25 kg/m^2^)	Overweight (25 kg/m^2^ ≤ BMI < 30 kg/m^2^)	Obese (BMI ≥ 30 kg/m^2^)	*P* value
Lesion (*n*)	586	458	108	20	
Operator, trainee (*n*)	327 (55.8%)	251 (54.8%)	64 (59.3%)	12 (60.0%)	0.653
Procedure time (min, median [IQR^*1^])	31.0 (20.0–53.0)	30.0 (20.0–50.8)	33.5 (22.0–60.0)	35.5 (24.5–48.0)	0.395
Longer procedure time (≥ 90 min) (*n*)	62 (10.6%)	42 (9.2%)	17 (15.7%)	3 (15.0%)	0.110
Specimen size (mm, median [IQR])	32.0 (24.0–40.0)	32.0 (25.0–40.0)	32.0 (26.0–40.0)	33.0 (24.0–41.3)	0.673
Lesion size (mm, median [IQR])	24.0 (20.0–30.0)	23.0 (20.0–30.0)	25.0 (20.0–32.0)	23.0 (16.0–32.5)	0.503
Use of hemostatic forceps (*n*)	53 (9.0%)	41 (9.4%)	9 (8.3%)	3 (15.0%)	0.627
Fibrosis (*n*)					0.878
F0	472 (80.5%)	368 (80.3%)	89 (82.4%)	15 (75.0%)	
F1	76 (13.0%)	59 (12.9%)	14 (13.0%)	3 (15.0%)	
F2	38 (6.5%)	31 (6.8%)	5 (4.6%)	2 (10.0%)	
*En bloc* resection (*n*)	585 (99.8%)	458 (100%)	107 (99.1%)	20 (100.0%)	0.109
Complete resection (*n*)	579 (98.8%)	454 (99.1%)	105 (97.2%)	20 (100.0%)	0.230
Curative resection (*n*)	573 (97.8%)	449 (98.0%)	104 (96.3%)	20 (100.0%)	0.430
Vertical margin positivity (*n*)	6 (1.0%)	4 (0.9%)	2 (1.9%)	0 (0.0%)	0.595
Horizontal margin positivity (*n*)	1 (0.2%)	0 (0.0%)	1 (0.9%)	0 (0.0%)	0.109
Tumor budding (*n*)					0.617
Grade 1	52 (8.9%)	40 (8.7%)	10 (9.3%)	2 (10.0%)	
Grade 2, 3	2 (0.3%)	2 (0.4%)	0 (0.0%)	0 (0.0%)	
Patients (*n*)	523	407	98	18	
Change of vital signs during ESD (*n*)					
SBP fluctuation^*2^	110 (21.0%)	88 (21.6%)	27 (27.6%)	5 (27.8%)	0.403
Bradycardia^*3^	30 (5.7%)	24 (5.9%)	5 (5.1%)	1 (5.6%)	0.905
Hypoxemia^*4^	176 (33.7%)	123 (30.2%)	43 (43.9%)	10 (55.6%)	0.005
Amount of flunitrazepam (mg/kg, median [IQR])	1.67 × 10^−2^ (1.22 × 10^−2^–2.27 × 10^−2^)	1.75 × 10^−2^ (1.27 × 10^−2^–2.34 × 10^−2^)	1.48 × 10^−2^ (1.08 × 10^−2^–2.03 × 10^−2^)	1.16 × 10^−2^ (0.98 × 10^−2^–1.54 × 10^−2^)	< 0.001
Amount of pethidine (mg/kg, median [IQR])	0.59 (0.51—0.69)	0.63 (0.55–0.72)	0.50 (0.46–0.56)	0.39 (0.32–0.45)	< 0.001
Delayed bleeding (*n*)	7 (1.3%)	7 (1.7%)	0 (0.0%)	0 (0.0%)	0.314
Perforation (*n*)	4 (0.8%)	3 (0.7%)	1 (1.0%)	0 (0.0%)	0.893
PECS^*5^ (*n*)	5 (1.0%)	3 (2.6%)	2 (2.0%)	0 (0.0%)	0.450
Delayed event^*6^ (*n*)					
Pulmonary embolism	2 (0.4%)	1 (0.2%)	1 (1.0%)	0 (0.0%)	0.518
Sick sinus syndrome	1 (0.2%)	0 (0.0%)	1 (1.0%)	0 (0.0%)	0.114
Subarachnoid hemorrhage	1 (0.2%)	1 (0.2%)	0 (0.0%)	0 (0.0%)	0.867
Hospitalization^*7^ (days, median [IQR])	5.0 (5.0–5.0)	5.0 (5.0–5.0)	5.0 (5.0–5.0)	5.0 (5.0–5.0)	0.927

^*^1 Interquartile range

^*^2 Systolic blood pressure fluctuation of more than 50 mm Hg

^*^3 Heart rate decreasing below 50 /min

^*^4 Oxygen desaturation below 90%

^*^5 Post-ESD coagulation syndrome

^*^6 Adverse events other than bleeding and perforation that occurred within 30 days after ESD

^*^7 Number of hospitalization days

^*^8 Body mass index

Table 3 presents the results of the present study limited to cases treated by trainees with less than 100 cases of colorectal ESD experience. The number of cases that required a procedure time of longer than 90 min was significantly larger in the group with a higher BMI (27 [10.8%] vs. 14 [21.9%] vs. 3 [25.0%], *P* = 0.033). No significant differences in the self-completion rates, *en bloc* resection rates, mean specimen sizes, mean lesion sizes, and pathological results were seen. The incidence of hypoxemia was not significantly different, but a higher BMI tended to increase the risk of hypoxemia (77 [34.8%] vs. 27 [48.2%] vs. 6 [54.5%], *P* = 0.096). Similar to the overall analysis, the amount of pethidine per body weight was significantly lower in the group with a higher BMI (0.61 [0.54–0.69] mg/kg vs. 0.49 [0.47–0.56] mg/kg vs. 0.38 [0.33–0.46] mg/kg, *P* < 0.001). No significant differences in complications or delayed events were seen.

**Table 3:** Clinical outcome of colorectal ESD performed by trainees according to body mass index groups

	Total	Control (BMI^*8^ < 25 kg/m^2^)	Overweight (25 kg/m^2^ ≤ BMI < 30 kg/m^2^)	Obese (BMI ≥ 30 kg/m^2^)	*P* value
Lesion (*n*)	327	251	64	12	
Procedure time (min, median [IQR^*1^])	40.0 (26.0–60.0)	40.0 (25.0–59.0)	47.0 (30.0–74.3)	38.0 (30.8–63.0)	0.150
Longer procedure time (≥ 90 min) (*n*)	44 (13.5%)	27 (10.8%)	14 (21.9%)	3 (25.0%)	0.033
Specimen size (mm, min, median [IQR])	30.0 (24.5–38.0)	30.0 (24.0–37.0)	30.0 (25.8–40.0)	29.5 (23.5–36.0)	0.456
Lesion size (mm mean ± SD)	22.0 (19.0–29.0)	22.0 (18.5–28.0)	24.5 (19.8–32.0)	20.0 (16.0–29.0)	0.274
Fibrosis (*n*)					0.942
F0	277 (84.7%)	214 (85.3%)	53 (82.8%)	10 (83.3%)	
F1	33 (10.1%)	24 (9.6%)	8 (12.5%)	1 (8.3%)	
F2	17 (5.2%)	13 (5.2%)	3 (4.7%)	1 (8.3%)	
Self-completion (*n*)	279 (85.3%)	218 (86.0%)	51 (79.7%)	10 (83.3%)	0.345
*En bloc* resection (*n*)	326 (99.7%)	251 (100%)	63 (98.4%)	12 (100.0%)	0.127
Complete resection (*n*)	322 (98.5%)	249 (99.2%)	61 (95.3%)	12 (100.0%)	0.070
Curative resection (*n*)	317 (96.9%)	245 (97.6%)	60 (93.8%)	12 (100.0%)	0.228
Vertical margin positivity (*n*)	4 (1.2%)	2 (0.8%)	2 (3.1%)	0 (0.0%)	0.295
Horizontal margin positivity (*n*)	1 (0.3%)	0 (0.0%)	1 (1.6%)	0 (0.0%)	0.127
Tumor budding (*n*)					0.862
Grade 1	29 (8.9%)	21 (8.4%)	6 (9.4%)	2 (16.7%)	
Grade 2, 3	1 (0.3%)	1 (0.4%)	0 (0.0%)	0 (0.0%)	
Patients (*n*)	288	221	56	11	
Change of vital signs during ESD (*n*)					
SBP fluctuation^*2^	77 (26.7%)	57 (25.8%)	17 (30.4%)	3 (27.3%)	0.788
Bradycardia^*3^	21 (7.3%)	16 (7.2%)	5 (8.9%)	0 (0.0%)	0.580
Hypoxemia^*4^	110 (38.2%)	77 (34.8%)	27 (48.2%)	6 (54.5%)	0.096
Amount of flunitrazepam (mg/kg, median [IQR]^*4^)	1.73 × 10^−2^ (1.27 × 10^−2^–2.26 × 10^−2^)	1.77 × 10^−2^ (1.32 × 10^−2^–2.29 × 10^−2^)	1.55 × 10^−2^ (1.17 × 10^−2^–2.23 × 10^−2^)	1.33 × 10^−2^ (1.21 × 10^−2^–1.98 × 10^−2^)	0.197
Amount of pethidine (mg/kg, median [IQR])	0.58 (0.51–0.66)	0.61 (0.54–0.69)	0.49 (0.47–0.56)	0.38 (0.33–0.46)	< 0.001
Delayed bleeding (*n*)	6 (2.1%)	6 (2.7%)	0 (0.0%)	0 (0.0%)	0.395
Perforation (*n*)	3 (1.0%)	2 (0.9%)	1 (1.8%)	0 (0.0%)	0.796
PECS^*5^ (*n*)	3 (1.0%)	1 (0.5%)	2 (3.6%)	0 (0.0%)	0.114
Delayed event^*6^ (*n*)					
Pulmonary embolism	1 (0.3%)	0 (0.0%)	1 (1.8%)	0 (0.0%)	0.125
Sick sinus syndrome	1 (0.3%)	0 (0.0%)	1 (1.8%)	0 (0.0%)	0.125
Hospitalization^*7^ (days, median [IQR])	5.0 (5.0–5.0)	5.0 (5.0–5.0)	5.0 (5.0–5.0)	5.0 (5.0–5.0)	0.721

^*^1 Interquartile range

^*^2 Systolic blood pressure fluctuation of more than 50 mm Hg

^*^3 Heart rate decreasing below 50 /min

^*^4 Oxygen desaturation below 90%

^*^5 Post-ESD coagulation syndrome

^*^6 Adverse events other than bleeding and perforation that occurred within 30 days after ESD

^*^7 Number of hospitalization days

^*^8 Body mass index

## Discussion

In this study, we analyzed the impact of obesity on the performance of colorectal ESD. No significant differences in short-term clinical outcomes, such as the *en bloc* resection rate, pathological results and adverse events, were seen between obese patients and non-obese patients. Although the impact of obesity on the performance of colorectal ESD has not been previously reported, the present study showed that ESD is safe and effective for obese patients. However, obesity was significantly associated with changes in the respiratory status during ESD.

A previous study showed that risk factors for hypoxemia associated with sedation during endoscopy were an older age, the presence of major comorbidities, the total pethidine dose, and obesity [21–24]. Although these factors have been reviewed for routine endoscopy, few reports have examined the risk of hypoxemia during colorectal ESD. In the present study, obese patients were shown to have a significantly increased incidence of hypoxemia during colorectal ESD. The presence of obstructive sleep apnea (OSA) is reportedly related to hypoxemia during the sedation of morbidly obese patients [25]. Although we did not examine the presence of OSA in this study, obesity is generally a risk factor for sleep apnea syndrome, which may have affected the present results.

In our hospital, if hypoxemia occurs during colorectal ESD, oxygen is first administered through a nasal cannula. In the cases with hypoxemia reported here, the oxygen saturation improved in all the cases with the administration of oxygen, and the administration of a sedative antagonist during the procedure or an emergency tracheal intubation was not required in any of the cases. In our hospital, anesthesiologists usually do not attend colorectal ESD procedures, and the sedation is performed by the endoscopists. However, the patient’s vital signs are constantly monitored by doctors and nurses other than the doctor performing the procedure, and sedatives are added as needed based on the patient’s condition. Since the amount of sedative was intentionally reduced in obese patients because of their susceptibility to hypoxemia, the amount of sedatives per kilogram of body weight thought to be significantly lower in the group with a higher BMI in this study.

On the other hand, when the procedures were performed by trainees, the number of cases that required a long procedure time was significantly larger in the group with a higher BMI. This result may reflect the difficulty of performing colorectal ESD in obese patients, particularly for trainees. In previous reports, predictors of colorectal ESD difficulty included a location in the right colon, tumor size, submucosal infiltration, fibrosis, non-experienced physician, and manipulation difficulties, but no previous report has shown a relationship with patient obesity [8, 15, 26, 27]. In these reports, scope manipulation difficulties were defined as either paradoxical movements, poor control because of adhesions, or the motion of the lesion as a result of heartbeats or breathing. In obese patients, previous studies have suggested that scope insertion can be difficult because of the formation of loops in the scope, difficulties in changing the patient’s position, ineffective abdominal pressure, and inadequate bowel preparation [28–30]. In addition, obese patients may experience sudden respiratory changes associated with OSA. The above factors are thought to be responsible for the poor scope maneuverability during ESD in obese patients. However, regarding the difficulty of insertion, in this study, no significant difference was seen among the groups in terms of the preoperative insertion time. In previous studies examining colonoscope insertion, not only obesity, but also being underweight was reported to be a factor in insertion difficulty [28–30]; therefore, the results for insertion time in the present study might also have been affected by patients who were underweight. Kang et al. reported that the procedure time was significantly longer in obese patients undergoing gastric ESD [10]. According to their study, a large amount of submucosal fat tissue in obese patients was thought to make dissection more difficult because of poor vision arising from burnt fat or aerosolized fat droplets; a similar situation might have occurred in the present study.

The procedure time for trainees operating on obese patients was thought to be prolonged in this study for the above reasons. Because the usefulness of overtube during ESD in the right-side colon, where scopes tend to form loops, has also been reported [31], the use of an overtube may be one option for performing ESD in the right-side colon of obese patients.

In our hospital, when a trainee performs ESD, an expert always supervises the procedure. As a result, no significant difference in the *en bloc* resection rate or the number of complications were seen in the present study. However, a long procedure time has been reported to be a risk factor for perforation [32]. In facilities where experts routinely observe procedures, the replacement of the physicians by the expert observing the procedure might be advisable if difficulties are anticipated so as to minimize the procedure time and reduce the risk of perforation.

Regarding delayed events, one patient developed sick sinus syndrome, two patients developed pulmonary embolisms, and one patient developed a subarachnoid hemorrhage. Although no significant differences were seen among the groups, some reports have described significantly higher risks of cardiovascular complications in obese surgical patients [1–6]. Therefore, further investigation is needed to determine the rate of cardiovascular complications in obese patients undergoing colorectal ESD.

The present study had some limitations. This was a retrospective, single-institution study, and most of the patients analyzed in this study were Japanese. Therefore, these results may not be applicable to other ethnicities. A multicenter, prospective study with a larger number of cases is needed to validate our results. Second, this study might not have assessed the effects of visceral fat accurately because BMI is not always correlated with the amount of fat in the body. In addition, since scope maneuverability and the degree of fatty tissue in the submucosa during ESD could not be studied retrospectively in this study, the impact of poor scope maneuverability and an abundance of fatty tissue on the treatment of obese patients remains unclear. In order to clarify the effects of these factors, further examination about the evaluation method of the degree of fatty tissue and the scope maneuverability are needed.

## Conclusions

In conclusion, colorectal ESD was safe and effective in obese patients. However, ESD in patients with obesity requires attention to changes in respiratory conditions and potential difficulties that might be encountered by trainees performing such procedure.

## Data Availability

The datasets used and/or analyzed during the current study are available from the corresponding author upon reasonable request.

## Abbreviations

ESD: Endoscopic submucosal dissection
BMI: Body mass index
SpO_2_: Percutaneous arterial oxygen saturation
OSA: Obstructive sleep apnea
